# Identification and Characterization of EIN3/EIL Transcription Factor Family Members in *Pinus massoniana* Lamb.

**DOI:** 10.3390/ijms252211928

**Published:** 2024-11-06

**Authors:** Wenya Yu, Xingyue Ren, Jingjing Zhang, Zichen Huang, Yulu Zhao, Mengyang Zhang, Sheng Yao, Kongshu Ji

**Affiliations:** 1 State Key Laboratory of Tree Genetics and Breeding, Nanjing Forestry University, Nanjing 210037, China; ya163163@163.com (W.Y.); yaosheng0817@163.com (S.Y.); 2 Key Open Laboratory of Forest Genetics and Gene Engineering of National Forestry and Grassland Administration, Nanjing 210037, China; 3 Key Laboratory of Forestry Genetics & Biotechnology of Ministry of Education, Nanjing Forestry University, Nanjing 210037, China; 4 Co-Innovation Center for Sustainable Forestry in Southern China, Nanjing Forestry University, Nanjing 210037, China

**Keywords:** *Pinus massoniana*, EIN3/EIL, transcription factor, stress response, expression pattern

## Abstract

Transcription factors refer to types of proteins that perform significant functions in the process of gene expression regulation. The ethylene insensitive 3/ethylene insensitive 3-like (*EIN3/EIL*) family, functioning as significant transcription factors regulating ethylene, plays a critical role in the growth and development of plants and participates in the plant’s response to diverse environmental stresses. *Pinus massoniana* is an excellent native tree with high economic and ecological value. However, the study of *EIN3/EIL* genes in gymnosperms, for instance, *P. massoniana,* is still relatively limited. In this research, four putative *EIN3/EIL* genes were identified in the transcriptome of *P. massoniana*. Bioinformatics analysis showed that *PmEIL* genes contain a highly conserved EIN3 domain and other structural features of acidic, proline-rich and glutamine-rich sites. The molecular evolution tree analysis demonstrated that the *EIN3/EIL* family was partitioned into three categories (A, B, and C), and the number, type, and distribution of conserved motifs grouped in one category were similar. The results of qRT-PCR indicated that the expression levels of *PmEIL* genes were markedly elevated in needles compared to other tissues. Through the analysis of expression patterns of the *PmEIL* genes under various stress treatments, it was found that the *PmEIL* genes could participate in plant hormone stimulation induction, osmosis, drought and other response processes. In addition, PmEIL is a nuclear localization protein. PmEIL1, PmEIL3, and PmEIL4 are transcriptional activators, while PmEIL2 is a transcriptional suppressor. This research provides a basis for further elucidating the function of EIN3/EIL transcription factors in growth, development and stress response of *P. massoniana*.

## 1. Introduction

Transcription factor (TF), often termed trans-acting factor, is a kind of protein that can bind to DNA in a sequence-specific manner and modulate transcription [1]. TFs perform significant regulatory functions in plant growth and development and responses to adverse conditions by specifically interacting with the cis-acting elements of genes to activate or suppress gene transcription [2]. According to their structural characteristics, the TF family comprises 58 types, such as WRKY [3], MYB [4], WOX [5] and so on. Among them, ethylene insensitive 3/ethylene insensitive 3-like (EIN3/EIL) belongs to a minor TF family in higher plants [6].

Ethylene (ET) is a small molecular gas plant hormone and one of the first plant growth regulatory substances to be established as plant hormones [7]. With the deepening of research, it has been found that ET not only significantly affects various processes of plant growth and development, but also exerts a function in dealing with diverse environmental stressors [8]. The response mechanism of plants to ET has achieved remarkable progress, among which EIN3/EIL proteins are one of the crucial TFs in ET signaling pathways [9]. In 1997, the *EIN3* gene and its homologues *Ein3-like1 (EIL1)* and *EIL2* were cloned from the *EIN3* mutant of the model plant *Arabidopsis* for the first time, which together regulate the expression of ET response genes [10]. EIN3/EIL TFs have been studied in many species. For example, identification has been reported in *Arabidopsis* [10], *Nicotiana tabacum* [11], *Hevea brasiliensis* [12] and other plants. EIN3/EIL is a protein that can bind to DNA, and has a nuclear localization specificity [13], characterized by a highly conserved amino acid sequence at the N-terminal, and a lower conserved C-terminal sequence compared to the N-terminal sequence [10]. Therefore, scholars have proposed that the functional diversity of EIL members primarily originates from the C-terminal site of the protein [14]. Although *EIN3/EIL* genes have undergone extensive investigation, the molecular mechanisms in perennial woody plants have not been adequately explored [15].

*EIN3/EIL* genes are extensively implicated in a variety of biological processes. *EIN3/EIL* genes are implicated in regulating the cascade reaction and activating other TFs [16]. For example, ET signal is transmitted to the EIN3/EIL family through the MAP kinase cascade pathway, and EIN3/EIL TFs transmission signal positively regulates downstream genes [17]. *EIN3/EIL* genes exert a critical function in modulating a plant’s responses to various hormone signals. For example, studies have shown that *EIN3/EIL1* regulates the innate immune response of *A. thaliana* by negatively inhibiting the metabolic pathway of salicylic acid [18]. In *Nicotiana tabacum* overexpressing *TEIL*, the flower pistil length was longer, and the stigma was slightly prominent [19]. *EIN3/EIL* genes can support in the resistance to biological stress. After silencing *TaEIL1* in wheat with virus-induced gene silencing technology, the expression of defense-related genes is up-regulated and the sucrose content is increased, which enhances the resistance of wheat to stick-rust [20]. 

*Pinus massoniana* Lamb. is a crucial timber species in the south of China, which is widely used and has high economic value. It can not only be used in papermaking, pillars, construction, furniture, decoration, transportation and other industries, but also plays an irreplaceable role in ecological environment construction, such as barren mountain greening [21]. During the process of growth and development, *P. massoniana* is exposed to multiple biological and abiotic stresses, such as drought, salinity, insects and so on [22]. EIN3/EIL TFs are crucial for numerous biological processes in plants [23], which underscores the importance of studying their functions in *P. massoniana*. Due to the lack of available reference genome resources for *P. massoniana*, members of the PmEIL TF family were identified through transcriptome analysis. Meanwhile, a systematic identification and analysis of PmEIL TF was carried out through bioinformatics methods. Furthermore, the expression profiles of the *PmEIL* gene family under diverse stress conditions were investigated. This research provides a theoretical foundation for future investigations into the function and mechanism of *EIL* genes in *P. massoniana*, and also helps with future research on the response of *P. massoniana* to environmental stressors.

## 2. Results

### 2.1. Identification of EIN3/EIL Genes in P. massoniana

By searching for the EIN3/EIL protein HMM in the transcriptomes of *P. massoniana* and eliminating duplicate sequences and sequences with incomplete conserved domains, a total of four hypothetical EIN3/EIL protein sequences were identified and named as PmEIL1-PmEIL4 according to the identification order (Table S1). The protein sequence length of PmEIL ranges from 630 to 669 amino acids (aa); the molecular weight ranges from 70940.78 to 74670.3 Da; the isoelectric point (pI) varies between 5.62 and 5.84; the instability coefficients ranged from 42.01 to 51.82, and the instability indices were all greater than 40, indicating that they were all unstable proteins; the aliphatic amino acid index ranged from 61.78 to 66.51; the Grand average of hydropathicity ranged from −0.743 to −0.827, with all hydrophilic indices being negative, which indicates that they were hydrophilic proteins (Table S2).

### 2.2. Phylogenetic Analysis of PmEIL Genes

**Figure 1:**
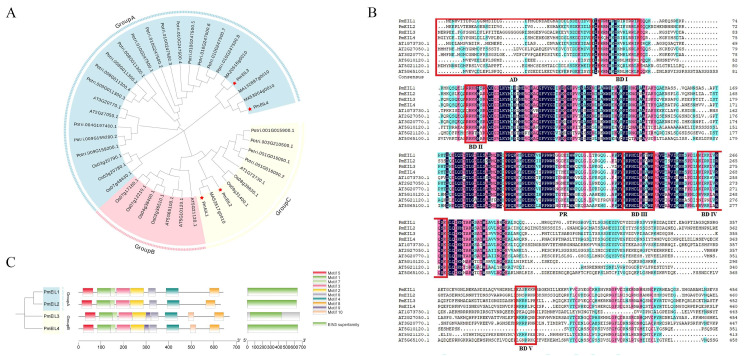
Phylogenetic analysis, conserved motif distribution, and structural domain characterization of EIN3/EIL proteins. (**A**) Phylogenetic classification of the EIN3/EIL family members of *P. massoniana*. Gymnosperm: *P. massoniana*, *P. abies*, *P. glauca*; Dicotyledon: *A. thaliana*, *P. trichocarpa*; Amphibrya: *O. sativa*, *Z. mays*. Red star symbolizes the EIN3/EIL protein of *P. massoniana*. The colors of the outermost circle signify different groupings; (**B**) Multiple sequence alignment of 10 EIN3/EIL family proteins in *P. massoniana* and *A. thaliana*. The distinct colors in the background indicate the differences in the degree of amino acid conservation within the sequence. The region demarcated by the red box indicates the acidic (AD) and basic domains (BDI-V) and the proline-rich domain (PR) present in the EIN3/EIL protein; (**C**) A total of ten conserved motifs within PmEIL proteins are displayed, with distinct colors denoting the different types of motifs.

To investigate the evolutionary diversity of EIN3/EIL proteins, seven phylogenetic trees containing monocos, dicots and gymnosperms were constructed based on EIN3/EIL amino acid sequences (Figure 1A). Generally speaking, the quantity of *EIL* genes is higher in monocots and dicots than gymnosperms. The 37 EIN3/EIL proteins included 4, 6, 9, 6, 7, 2 and 3 EIN3/EIL protein sequences of *P. massoniana*, *A. thaliana*, *Oryza sativa*, *Zea mays*, *Populus trichocarpa*, *Picea glauca* and *Picea abies*, respectively. According to phylogenetic analysis, the EIN3/EIL sequences can be classified into three subgroups, which are designated as Group A, B, and C. The number of *EIL/EIN3* genes in Group A amounts to 16, topping all the groups, while Group B and Group C contain 12 and 9 genes, respectively. The evolutionary relationship showed that there were two EIN3/EIL proteins of *P. massoniana* in group A and group B respectively, which were clustered in the same evolutionary cladism as *P. abies* and *P. glauca*, indicating that the EIN3/EIL TFs of gymnosperm were similar in evolutionary history. 

### 2.3. Sequence Alignment and Motif Analysis of PmEIL

Employing DNAMAN 6.0 software, multiple sequence alignment was conducted for the 10 protein sequences belonging to the EIN3/EIL TFs family of *P. massoniana* and *A. thaliana*. It was discovered that the EIN3/EIL protein sequences were highly conserved (Figure 1B). The results showed that PmEIL had the acidic amino acid domain (AD), alkaline amino acid domain (BDI-V) and proline-rich domain (PR) of *A. thaliana* EIN3/EIL, and the N-terminal of AD mainly contained Aspartic acid (A) and Glutamic acid (E); BDI-V is mainly composed of Arginine (R), Histidine (H) and Lysine (K); PR mainly contains proline (Pro-line, PR). Furthermore, acidic, proline-rich and glutamine-rich regions are often reported as typical transcriptional activation domains in plants, indicating that these rich regions perform a crucial function in the transcriptional activation and functional regions of the EIN3/EIL TF family. 

Through MEME website, 10 conserved motifs were predicted in PmEIL and were respectively labeled as Motif 1 to Motif 10 (Figure 1C). The 10 motif amino acids range in length from 20 to 60 (Table S3). Among the conserved motifs of PmEIL, only motif 1 - motif 3 and motif 7 are associated with the EIN3/EIL (PF04873) domain and are positioned in the first half of the amino acid sequence. It is noteworthy that the motifs’ distribution aligns with evolutionary classification. Members within the same clade exhibit identical motif compositions, suggesting that PmEIL members grouped within the same subclass might possess similar functions.

### 2.4. Subcellular Localization of PmEIL 

**Figure 2:**
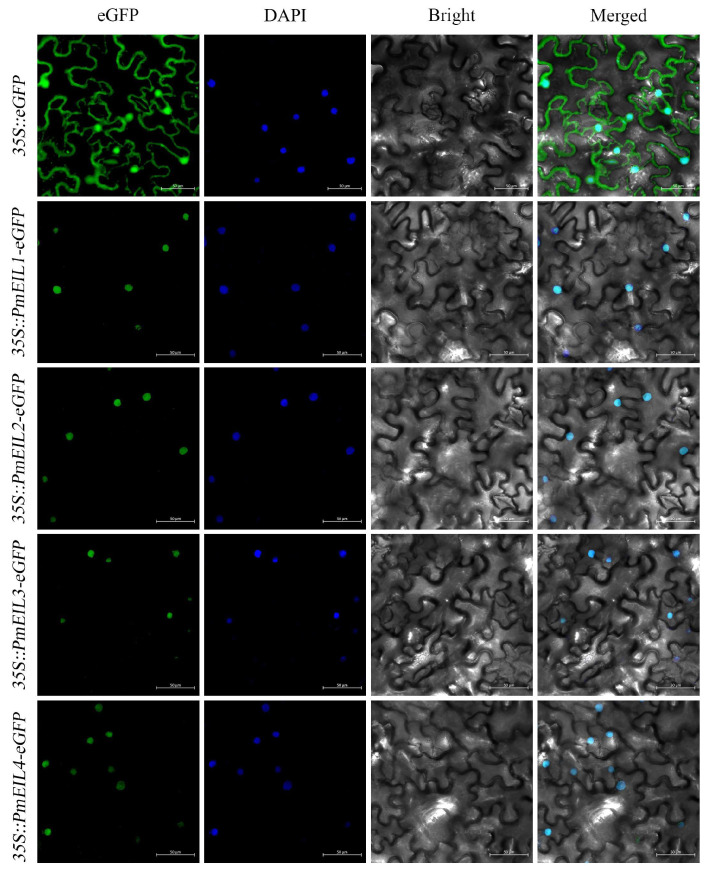
Subcellular localization experiments of PmEIL proteins. Transient expression of 35S::eGFP (control), 35S::PmEIL-eGFP in *N. benthamiana* leaves. Scale bar = 20 µm.

The Cell-PLoc 2.0 website predicts that all PmEIL proteins are situated in the nucleus (Table S4). In order to confirm its characteristics of subcellular localization, transient transformation experiments were performed. Fluorescence signals were discerned in the leaves of *N. benthamiana* that underwent instantaneous transformation (Figure 2). Through examination of the illustration, eGFP signals in the control group were distributed throughout the cell, whereas eGFP fused with PmEIL showed fluorescence only in the nucleus. The above research shows that all PmEIL TFs are proteins with nuclear localization.

### 2.5. Transcription Profile Analysis of PmEIL

**Figure 3:**
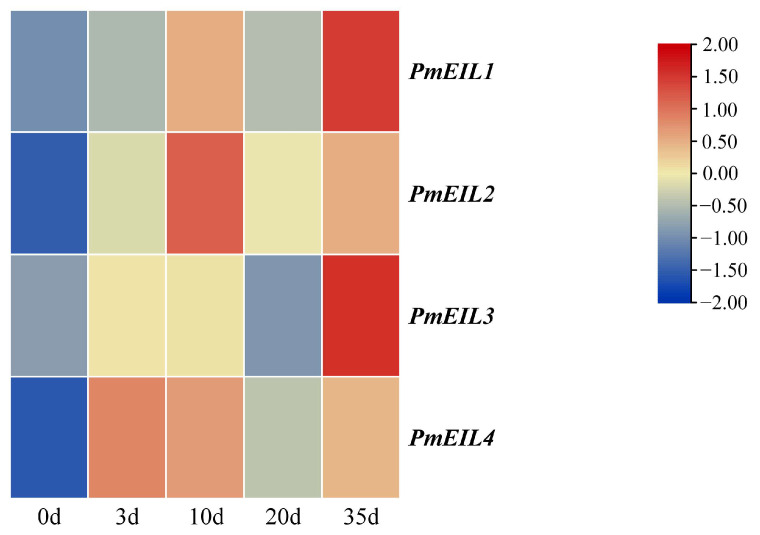
Transcriptional patterns of PmEIL family members in *P. massoniana* following inoculation with pinewood nematodes across five distinct stages: 0 (CK), 3, 10, 20, and 35 d. A heatmap is constructed based on the log_2_(FPKM + 1) values along with row normalization. The color gradient indicates the relative expression level, with red denoting high expression and blue denoting low expression.

To assess the potential impact of the *PmEIL* genes on pine wood nematode (PWN) inoculation, based on the transcriptomics data (SRA accession: PRJNA66087) previously obtained by our research group, we analyzed the expression pattern of the *PmEIL* genes and constructed an expression heatmap (Figure 3). The findings indicated that *PmEIL1-PmEIL4* genes were detected and expressed after inoculation. The expression of *PmEIL* genes exhibited an initial increase during inoculation, then decreased at 20 days after inoculation, and then increased again, showing an overall upward trend.

### 2.6. Tissue Expression Patterns of PmEIL Genes

**Figure 4:**
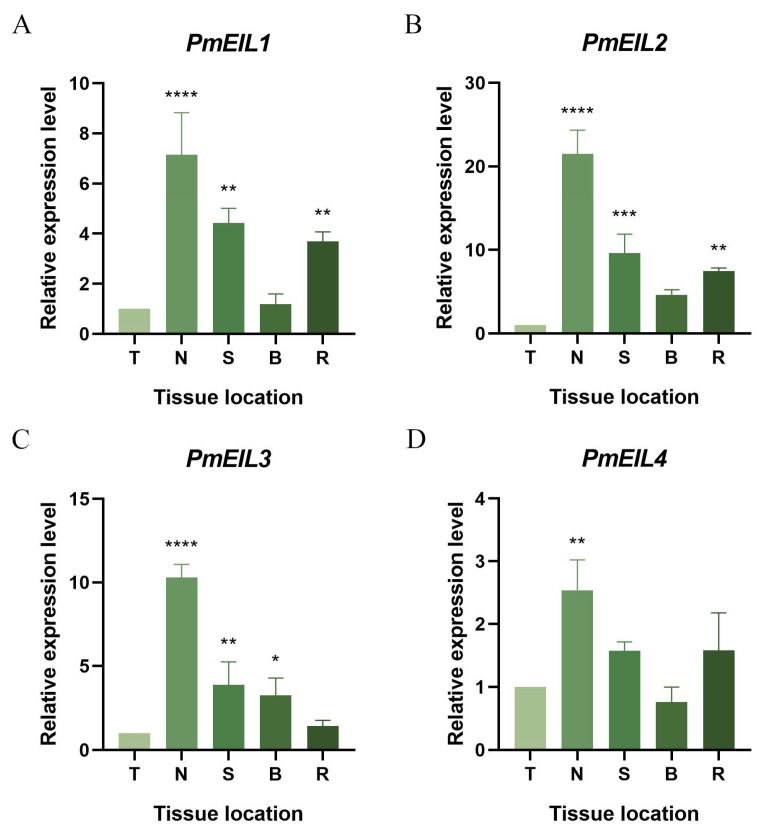
Analysis of the expression of *PmEIL* genes in various tissue sites. (**A**) *PmEIL1*; (**B**) *PmEIL2*; (**C**) *PmEIL3* (**D**) *PmEIL4.* T: terminal bud; N: needles; S: stems; B: bark; R: roots. The asterisks denote a statistically significant difference in transcript abundance between the treatment group and the control group (* *p* < 0.05, ** *p* < 0.01, *** *p* < 0.001, **** *p* < 0.0001).

The expression patterns of *PmEIL* genes in five distinct tissues of *P. massoniana* were analyzed and examined using qRT-PCR analysis (Figure 4). We found that *PmEIL1-PmEIL4* was expressed in terminal bud, needle, stem, bark, and root, but the expression levels were different. The expression levels of *PmEIL1* in needles, stems and roots were 7.2 times, 4.4 times and 3.7 times that of terminal bud, respectively (Figure 4A). The expression of *PmEIL2* in needles, stems and roots was significantly elevated compared to other tissues, being 21.5 times, 9.6 times and 7.5 times higher than that in terminal bud, respectively (Figure 4B). The expression levels of *PmEIL3* in needles, stems and bark were 10.3 times, 3.9 times and 3.3 times of that of terminal bud, respectively (Figure 4C). The expression of *PmEIL4* in other tissues was not significant, but the highest level in needles was 2.5 times that of terminal bud (Figure 4D). In conclusion, the expression levels of *PmEIL1-PmEIL4* in needles were conspicuously higher than that in other tissues, which suggested that *PmEIL* was mainly expressed in needles.

### 2.7. Expression Patterns of PmEIL Genes Under Different Treatments

**Figure 5:**
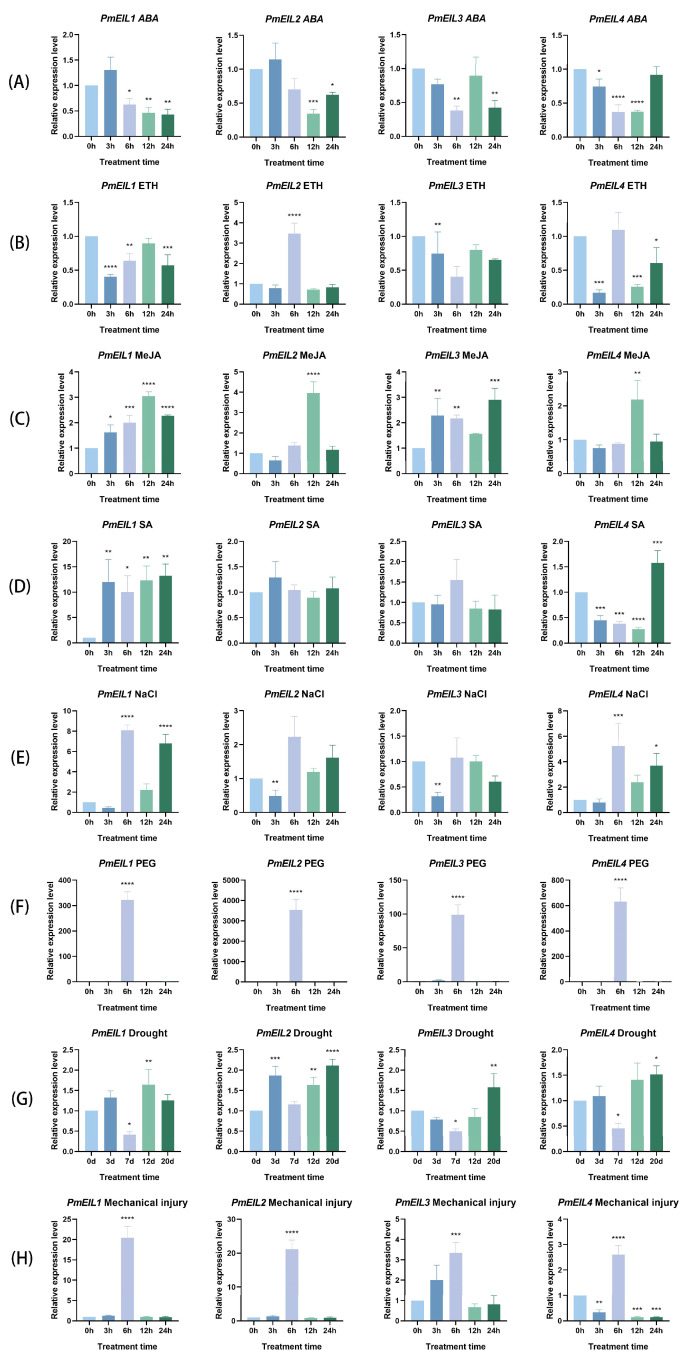
The expression patterns of *PmEIL* genes under various treatments. (**A**) ABA; (**B**) ETH; (**C**) MeJA; (**D**) SA; (**E**) NaCl; (**F**) PEG; (**G**) Drought; (**H**) Mechanical injury. The asterisks signify statistically significant differences in transcript abundance between the treatment group and the control group (0 h) (* *p* < 0.05, ** *p* < 0.01, *** *p* < 0.001, **** *p* < 0.0001).

The expression of the *PmEIL* gene responses were evaluated under eight different abiotic stresses (Figure 5). Under ABA conditions, the expressions of *PmEIL1* and *PmEIL3* were conspicuously decreased at 6 h, *PmEIL2* and *PmEIL4* decreased respectively at 12 h and 3 h (Figure 5A). In the presence of ETH, the expressions of *PmEIL1* and *PmEIL3* were the lowest at 3 h, the expression of *PmEIL4* began to decline at 3 h, then increased and then decreased, while the expression of *PmEIL2* only reached its peak at 6 h (Figure 5B). After MeJA treatment, *PmEIL1*, *PmEIL2* and *PmEIL4* increased first and then decreased, and the expression of *PmEIL3* reached its maximum level at 12 h, while the expression of *PmEIL3* reached its maximum level at 24 h (Figure 5C). Following SA treatment, the expression of *PmEIL1* increased, the expression of *PmEIL4* decreased and then increased significantly, while *PmEIL2* and *PmEIL3* did not change significantly, indicating that they were not sensitive to SA treatment (Figure 5D). Under conditions of NaCl, the expression levels of *PmEIL1* and *PmEIL4* increased notably at 6 h, decreased at 12 h, and increased again at 24 h. The expressions of *PmEIL2* and *PmEIL3* were the lowest at 3 h (Figure 5E). Under conditions of PEG, the expression of *PmEIL1-PmEIL4* reached its peak at 6 h, and compared with other stresses, the expression level of *PmEIL* genes under PEG stress was the highest overall (Figure 5F). Under treatment of drought, the expression levels of *PmEIL1*, *PmEIL3* and *PmEIL4* decreased remarkably at 7 d, and then continued to increase; *PmEIL2* increased significantly at 3 d, then decreased, and reached a peak at 20 d (Figure 5G). Under the conditions of mechanical injury, the expression of *PmEIL1-PmEIL4* reached its peak at 6 h, and the overall expression levels showed a downward trend in the process (Figure 5H). In summary, *PmEIL* genes can respond to different treatment conditions, suggesting that they might perform an important function where *P. massoniana* is subjected to various abiotic stresses. Furthermore, the expression pattern of *PmEIL* in response to multiple stresses is different, which suggests that *PmEIL* genes might exhibit distinct response mechanisms in resisting different abiotic stresses in *P. massoniana*.

### 2.8. Transcriptional Activity Analysis of PmEIL2 and PmEIL4

**Figure 6:**
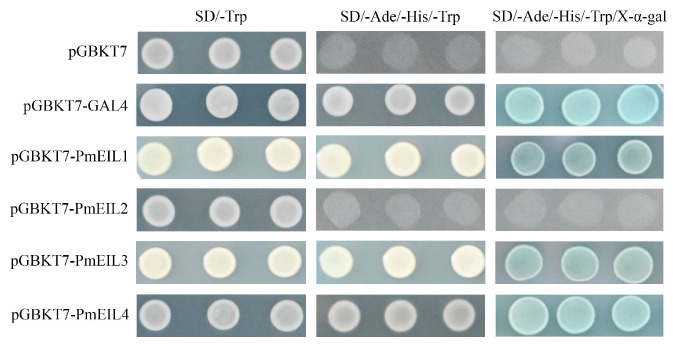
Transcriptional self-activation assay for pGBKT7-PmEIL vector. pGBKT7: negative control; pGBKT7-*GAL4*: positive control.

Additionally, we measured the transcriptional activity of these four PmEIL proteins (Figure 6). The findings indicated that the yeast cells carrying the fusion vector of pGBKT7-*PmEIL1,* pGBKT7-*PmEIL3* and pGBKT7-*PmEIL4* can grow on SD/-Ade/-His/-Trp medium, and exhibited a blue coloration in the presence of the chromogenic substrate X-α-gal. Other yeast cells containing the recombinant plasmid had the same negative control results as shown in the figure, and were unable to grow on the SD/-Ade/-His/-Trp medium. The above results indicate that PmEIL2 proteins belong to transcriptional suppressors, while PmEIL1, PmEIL3 and PmEIL4 proteins belong to transcriptional activators, which lays a foundation for further research on gene regulation.

## 3. Discussion

EIN3/EIL has been identified in many species, and the quantity of its family members varies greatly between species, with 17 *EIN3/EIL* genes identified in banana [24], 10 in sweet orange [15] and 5 in tobacco [11]. Research indicates that EIN3/EIL TFs play significant roles in the integration hub of ET and other signals, thus regulating the growth and development of plants and responding to different environmental stresses [25]. Therefore, the research on the EIN3/EIL TFs in *P. massoniana* holds great importance. In this research, EIN3/EIL TFs were identified by four *P. massoniana* transcriptomes and comprehensively analyzed.

A total of four EIN3/EIL TFs were screened from the transcriptome of *P. massoniana* and designated as *PmEIL1-PmEIL4*. Through phylogenetic analysis, the *EIN3/EIL* family of *P. massoniana* can be classified into three categories. The findings demonstrated that not all *PmEIL* genes were categorized into the same class, indicating that functional disparities might exist among these members. Multiple sequence alignment results showed that all PmEIL proteins possess a highly conserved EIN3 domain, which is a characteristic structure of EIN3/EIL [12]. The PmEIL proteins likewise demonstrated the significant structural characteristics of EIN3/EIL, encompassing a highly acidic N-terminal amino acid region, basic amino acid region and proline-rich region [25]. This indicated that this gene family might possess certain similar functions in higher plants. Through conservative motif prediction, it was revealed that the quantities and types of conserved motifs in the PmEIL proteins of the same group were analogous, and the distribution sequence was also uniform. This provides compelling evidence for the phylogenetic evolutionary relationships of PmEIL, and concurrently implies functional disparities among the members. 

Proteins are localized in the appropriate region to be able to perform their function. PmEIL was found to be a nuclear localization protein, based on amino acid sequence analysis and verified by tobacco transient transformation test, which is consistent with the location of the *EIL* genes in *H. brasiliensis* [14] and *Brassica napus* [26]. The results are in accordance with our common understanding that TFs are usually within the nucleus of cells. 

Patterns of gene expression are closely associated with the functions of genes. In the EIN3/EIL1 double mutant of *A. thaliana*, ET has the ability to stimulate the salicylic acid signaling pathway in conjunction with various TFs, thereby contributing to a defense response to *P. brassicae* infection [27]. The elimination of *TaEIL1* can slow down the growth of *Puccinias triiformis* f. sp. *Tritici* Eriks. and enhance the resistance of *Triticum aestivum* to Wheat Stripe Rust [28]. Based on transcriptome data (SRA login: PRJNA66087), we observed the transcriptional abundance of the *PmEIL* gene during PWN resistance. The research findings indicated that the expression quantity of the *PmEIL* genes increased significantly after PWN infection. Consequently, it can be hypothesized that *PmEIL* genes play a crucial role in the process of *P. massoniana* resisting PWN, laying a foundation for subsequent functional studies.

In higher plants, EIN3/EIL TFs are found in nearly all types of tissues, but their expression levels and patterns are tissue specific. The expression level of *CsEIL1*-*CsEIL8* in sweet orange leaves was significantly higher than the relative expression level, suggesting that it could be implicated in the growth and development of leaves [15]. *OsEIL1* exhibits a high expression level in the taproot of rice and can be involved in the process of ET-induced inhibition of taproot growth [28]. In order to explore the expression characteristics of *PmEIL* in diverse tissues of *P. massoniana,* the expression of *PmEIL* was analyzed. The results demonstrated that *PmEIL* genes were expressed in all tissue sites of *P. massoniana*, but the expression in the needles was conspicuously higher than that in other tissues. These results suggest that the *PmEIL* gene may be related to needle growth and development.

EIN3/EIL TFs have been verified to engage in the modulation of plant hormones and other stress reactions [29]. *A. thaliana* inhibited the seed germination of EIN3-EIL1 double mutant under high salt stress, while the germination rate was markedly enhanced in the *EIN3* overexpression strain, resulting in improved salt tolerance among the seedlings, suggesting that *EIN3* gene expression could enhance germination, seedling growth and salt resistance [30]. ET, through its signaling core transcription factor *OsEIL1*, activated the expression of gibberellin metabolism gene *OsGA2ox1/2/3/5* to reduce the content of active gibberellin in roots, thereby inhibiting cell proliferation in root meristem and ultimately inhibiting the growth of initial roots of rice seedlings [31]. Overexpression of *MnEIL3* in *A. thaliana* results in enhanced tolerance to salt stress and drought stress, suggesting that *MnEIL3* could have a crucial influence on the expression of abiotic stress tolerance and ET biosynthesis genes in mulberry [32]. Consequently, it is of great significance to observe the expression pattern of *PmEIL* genes through various stress treatments. Upon exogenous hormone treatment, the expression level of *PmEIL* underwent significant alterations, and it is speculated that *PmEIL* might provide a function in the hormone-mediated signaling pathways. This establishes the groundwork for further investigations into the function of *PmEIL* in hormone signal transduction in the future. The *PmEIL* genes show significant upregulation to varying extents under osmotic stress, drought, and mechanical injury circumstances, suggesting that these genes might possess a conserved function in the response of *P. massoniana* to external stimuli via positive regulation. The foregoing results indicate that the *PmEIL* genes are likely to play an essential role in the response of *P. massoniana* to external environmental stress. 

Transcriptional activation activity is related to whether EIN3/EIL can activate downstream reporter genes. Therefore, through scrutinizing the growth attributes of yeast cells bearing the pGBKT7-PmEIL fusion expression vector under nutrient-deficient circumstances, it was proven that *PmEIL2* could not grow normally, whilst the remaining PmEIL genes exhibited transcriptional activation activity. The findings established a foundation for the subsequent determination of yeast dihybrid, and provided a basis for the study of EIN3/EIL protein related regulatory mechanisms [33].

In conclusion, this research conducted a thorough and systematic analysis of the EIN3/EIL TFs of *P. massoniana*, which is highly conserved in the evolutionary protocol and plays a vital role in different abiotic stress responses. The study results contribute to further understandings of the dynamic regulation of *EIN3/EIL* gene regulatory network abiotic stress conditions in *P. massoniana*.

## 4. Materials and Methods

### 4.1. Identification of EIN3/EIL TFs

To identify the EIN3/EIL family members present in the *P. massoniana* transcriptomes, the Hidden Markov model (HMM) profile was first obtained in accordance with the specific conserved structural domain (PF04873) of the EIN3/EIL TFs from the Pfam database (https://pfam.xfam.org/, accessed on 27 October 2023). The HMM profile was used to search the EIL protein members in the *P. massoniana* transcriptomes by the HMME 3.0 software, and the threshold was set to E < 10^−5^. The transcriptomes data of *P. massoniana* were derived from drought stress transcriptome (SRA accession: PRJNA595650) [34], CO_2_ stress transcriptome (SRA accession: PRJNA561037) [35], tender shoots transcriptome (SRA accession: PRJNA655997) and *P. massoniana* inoculated with PWN transcriptome (SRA accession: PRJNA66087) [36]. To further substantiate the validity of these candidate sequences, the online website SMART (http://smart.embl-heidelberg.de/, accessed on 27 November 2023) and the CD in the NCBI-Search (https://www.ncbi.nlm.nih.gov/Structure/cdd/wrpsb.cgi, accessed on 28 November 2023) was used to predict the candidate EIN3/EIL protein domain structure. Subsequently, incomplete domains and protein sequences with a similarity exceeding 97% were eliminated. Protein sequences obtained by screening are shown in Table S1.

### 4.2. Sequence Analysis and Phylogenetic Analysis

The physicochemical properties of the *P. massoniana* EIL/EIN3 protein were analyzed, employing the ExPASy website (https://web.expasy.org/protparam/, accessed on 2 December 2023), including its theoretical molecular weight, isoelectric point (pI), instability coefficient, aliphatic amino acid index, and hydrophilicity index. Following the acquisition of the protein sequences for EIN3/EIL from *A. thaliana*, the protein sequences for *P. trichocarpa*, *O. sativa*, *Z. mays*, *P. glauca* and *P. abies* were obtained from the Plant Transcription Factor Database (https://planttfdb.gao-lab.org/, accessed on 10 December 2023), and multiple sequence alignments of EIN3/EIL member amino acid sequences were generated using the default parameters of the ClustalW program. Subsequently, the phylogenetic tree was constructed by employing the Neighbor-Joing (NJ) method within the MEGA (v10.2.6) software (Parameters are set to Poisson model and 1000 bootstraps) [37]. Furthermore, the phylogenetic tree was embellished using the EvolView website (https://www.evolgenius.info/evolview-v2/, accessed on 12 December 2023).

### 4.3. Sequence Alignment and Analysis of Conserved Motif 

Multiple sequences of EIN3/EIL proteins were compared between *P. massoniana* and *A. thaliana* using DNAMAN 6.0 software. The MEME platform (https://meme-suite.org/meme/, accessed on 24 December 2023) was utilized to analyze conserved motif distribution in PmEIL proteins and retrieve 10 motifs based on default parameters [38]. Subsequently, visualization and analysis were conducted using the Simple BioSequence Viewer plug-in incorporated in TBtools (v2.067) software.

### 4.4. Subcellular Localization of PmEIL Proteins

The online site Cell-PLoc 2.0 (http://www.csbio.sjtu.edu.cn/bioinf/Cell-PLoc-2/, accessed on 28 December 2023) was utilized to predict PmEIL proteins’ subcellular localization. The open reading frames (ORF) of *PmEIL1*-*PmEIL4* were obtained by gene cloning, and transient transformation experiments were performed. The primers employed for gene cloning and vector construction are displayed in Table S5. The ORF regions without termination codon were linked to the pCAMBIA1302-eGFP vector, and the 35S::*PmEIL*-eGFP expression vector was constructed with the recombinant enzyme, which was transformed into the *Agrobacterium* GV3101. Meanwhile, the P19 (RNA Silences Inhibitor) *Agrobacterium* strain was propagated at 28 °C for 36 h. It was then suspended in a solution containing 10 mM MgCl_2_, 150 μM Acetosyringone and 10 mM 2-Morpholinoethanesulfonic acid. After the suspended cells were combined with P19 in a 1:1 ratio, the mixed solution was injected into the leaves of *N. benthamiana* plants. The eGFP signal was acquired by an LSM710 confocal laser scanning microscope after 48 h dark culture of injected *N. benthamiana* plants.

### 4.5. Transcriptional Pattern Analysis

Through the bark inoculation experiment method, the research group simulated the infection of PWN in *P. massoniana*, and then collected the needles of the infected trees on days 0, 3, 10, 20, and 35. RNA sequencing data were obtained from the transcriptome of the infected *P. massoniana* (SRA accession: PRJNA66087), which were exploited to examine the expression of the *PmEIL* genes. The abundance of *PmEIL* transcripts was assessed by computing the number of base fragments per kilobase (FPKM) mapped by the exon model per million readings. The FPKM values are depicted in Table S6. TBtools (v2.067) software constructed heat maps derived from the values of log_2_ (FPKM + 1) and evaluated them on a row-scale [39].

### 4.6. Plant Materials and Treatments

One year old *P. massoniana* seedlings were provided by the State Key Laboratory of Forest Genetics and Breeding (Nanjing Forestry University). The seedlings were planted in nutrient soil (perlite: vermiculite: peat, 1:1:3) and cultured under 25℃ with a photoperiod of 16 h light and 8 h dark. After one month of growth of *P. massoniana* seedlings, the seedlings with good growth and consistent states were selected for follow-up treatment. To investigate the expression levels of *PmEIL* gene, five representative tissues or organs were selected, including terminal bud (T), needle (N), stem (S), bark (B), and root (R). Additionally, the seedlings were treated with the following eight methods: 50 μM ethephon (ETH), 10 mM methyl jasmonate (MeJA), 100 μM abscisic acid (ABA) and 1 mM salicylic acid (SA) were independently sprayed on the needles of the selected seedlings for hormone treatment [40,41]; 200 mM NaCl solution and 15% polyethylene glycol (PEG6000) [41,42] solution were applied to the soil to induce osmotic stress on the plants; cutting of the upper part of the needle, which caused mechanical damage; after watering at 0 days, the natural evaporation within 20 days caused drought stress to the plants. Under drought stress, needles were sampled at 0 d, 3 d, 7 d, 12 d and 20 d, and samples at 0 d were taken as control. Needles were sampled at 0 h, 3 h, 6 h, 12 h and 24 h after treatment in other stress experiments, and samples at 0 h were taken as control [43]. The obtained samples were rapidly frozen in liquid nitrogen and subsequently preserved at -80 °C. All of the above treatments were repeated by three separate biologists.

### 4.7. Total RNA Extraction, and Expression of the PmEIL Genes Analyzed by QRT-PCR

The extraction of total RNA was carried out by the FastPure Universal Plant Total RNA Isolation Kit (RC411-01, Vazyme Biotech, Nanjing, China). The concentration and purity of RNA were measured by NanoDrop2000 (Thermo Fisher Scientific, Waltham, MA, USA) instruments, and RNA integrity was measured using 1.2% agarose-gel electrophoresis. The First Strand cDNA synthesis kit (AT311, TransGen Biotech, Beijing, China) synthesized cDNA (20 μL) by reverse transcription of total RNA (1000 ng). QRT-PCR primers were designed using Primer 5.0, with a-tubulin (*TUA*) as the internal reference gene [44]. Primers are shown in Table S5. QRT-PCR was conducted with SYBR Green real-time PCR master mix (QPK-201, Toyobo Bio-Technology, Shanghai, China), and performed on the StepOne Plus device (Foster City Applied Biosystems, Foster City, CA, USA). The 10 μL system of each PCR mixture consisted of 5 μL of SYBR Green real-time PCR master mix, 1 μL of diluted cDNA (20× dilution), 0.4 μL of each primer and 3.2 μL of ddH_2_O. The following six steps constitute the PCR program: pre-denaturation at 95°C for 60 s; denaturation at 95 °C for 15 s, annealing at 60 °C for 15 s, and extension at 72°C for 10 s (40 cycles); melting at 95 °C for 0.5 s and annealing at 60 °C for 1 min. Three independent technical repetitions were performed for each reaction. The relative expression level of *PmEIL* gene was ascertained by employing the 2^−∆∆Ct^ method. Finally, the difference was analyzed through one-way ANOVA and multiple comparison tests with the GraphPad Prism 8.0 software.

### 4.8. Transcriptional Activity of Candidates

For the purpose of evaluating the transcriptional activation potential of PmEIL, the ORF of *PmEIL2* and *PmEIL4* genes were linked to the pGBKT7 vector. The details of the primers necessary for vector construction can be found in Table S5. The positive control plasmid pGBKT7-*GAL4*, negative control plasmid pGBKT7, and the recombinant plasmids pGBKT7-*PmEIL* were successfully transfected into the *AH109* yeast strain (YC1010, Weidi Biotech, Shanghai, China) and then cultivated on a tryptophan-deficient (SD/-Trp) medium at 28°C for 3 days. Subsequently, we selected the individual colonies that had been successfully transformed and diluted them with ddH₂O. We pipetted 5 microliters of the diluted solution and inoculated it onto the surfaces of the yeast media SD/-Trp and SD/-Trp/-Ade/-His, as well as the SD/-Trp/-Ade/-His medium containing X-α-gal, and cultured them at 28 °C. Finally, we observed the growth status of the yeast cells.

## 5. Conclusions

In this research, the EIN3/EIL TFs within *P. massoniana* were systematically identified and categorized for the first time. The results demonstrated that four candidate *PmEIL* genes were identified within the four transcriptome databases of *P. massoniana* and divided into 2 classes. Members of an aggregation class have the same number of similar motifs, indicating that they may have similar functions. PmEIL was confirmed as a nuclear localization protein by bioinformatic prediction and a subcellular localization test. Meanwhile, transcription auto activation tests showed that PmEIL2 was a transcriptional suppressor and the rest of the PmEIL were transcriptional activators. The expression pattern of the *PmEIL* genes undergoes alterations with the passage of time under various stress treatment conditions, which suggests the sensitivity of *PmEIL* genes to stress responses and reflects its potential as a candidate gene for future research on abiotic stress reactions. This research offers a theoretical foundation for the subsequent exploration of the functions of EIN3/EIL TFs. Meanwhile, as the initial report of the EIL genes in *P. massoniana*, the expression pattern of the *PmEIL* genes is conducive to further investigations into the role of *P. massoniana* in stress responses, and offers potential tactics for the molecular breeding of *P. massoniana* in the future.

## Data Availability

The data presented in this study are available in Supplementary Materials.

## Supplementary Materials

The supporting information can be downloaded at: https://www.mdpi.com/article/10.3390/ijms252211928/s1.
